# Reflections on the use of living animals in vet education through the creation of physiology didactic biomodels

**DOI:** 10.29374/2527-2179.bjvm003026

**Published:** 2026-06-12

**Authors:** David Fernando Balaguera Quinche, Rodrigo Forero Carrillo, Paula Andrea Balaguera Quinche

**Affiliations:** 1 School of Veterinary Medicine, Fundación Universitaria Agraria de Colombia, Bogotá, Colombia.; 2 Department of Basic Sciences, National University of Colombia, Bogotá, Colombia.

**Keywords:** biomodels, animals, physiology, teaching, veterinary, biomodelos, animais, fisiologia, ensino, veterinária

## Abstract

The use of living animals in education remains a topic of debate, particularly in health and veterinary sciences. While some authors justify animal experimentation as essential for scientific advancement and practical learning, others question its ethical implications and advocate for alternative teaching methods. This study aimed to explore veterinary students’ reflections on the use of living animals in physiology teaching through the development of functional didactic biomodels simulating physiological processes originally identified through experimental animal procedures.The study was conducted at two universities in Bogotá, Colombia, during a physiology course over one academic year. Initially, students participated in discussions addressing the history of animal experimentation and the major physiological discoveries obtained through invasive procedures involving animals. Subsequently, students developed three-dimensional didactic biomodels representing physiological mechanisms previously investigated through animal experimentation. At the end of the activity, students participated in open-ended experiential feedback sessions regarding the possibility of using living animals for educational purposes.The findings demonstrated heterogeneous perspectives among participants, ranging from acceptance to rejection of the practice. Some students opposed the use of animals based on ethical concerns and animal welfare principles, emphasizing the importance of alternative teaching approaches. Others recognized the educational value of alternative methods capable of replacing animal use. In contrast, some participants supported the use of living animals, arguing that direct contact with biological material provides a unique and impactful learning experience and may facilitate the integration of theoretical and practical knowledge, as well as stimulate future scientific research.

## Resumo

O uso de animais vivos na educação permanece um tema de debate, especialmente nas ciências da saúde e nas ciências veterinárias. Enquanto alguns autores consideram a experimentação animal essencial para o avanço científico e o aprendizado prático, outros questionam suas implicações éticas e defendem métodos alternativos de ensino. Este estudo teve como objetivo explorar as reflexões de estudantes de medicina veterinária sobre o uso de animais vivos no ensino de fisiologia, por meio do desenvolvimento de biomodelos didáticos funcionais que simulavam processos fisiológicos originalmente identificados por meio de procedimentos experimentais em animais. O estudo foi realizado em duas universidades de Bogotá, Colômbia, no âmbito de uma disciplina de fisiologia ao longo de um ano acadêmico. Inicialmente, os estudantes participaram de discussões sobre a história da experimentação animal e as principais descobertas fisiológicas obtidas por meio de procedimentos invasivos. Em seguida, desenvolveram biomodelos tridimensionais que representam mecanismos fisiológicos previamente investigados por meio de experimentação animal. Ao final da atividade, os participantes realizaram sessões abertas de feedback sobre a possibilidade do uso de animais vivos para fins educacionais. Os resultados evidenciaram perspectivas heterogêneas entre os estudantes. Parte dos participantes se opôs ao uso de animais devido a preocupações éticas e princípios de bem-estar animal, defendendo métodos alternativos de ensino. Outros reconheceram o potencial educacional dessas abordagens substitutivas. Em contrapartida, alguns estudantes apoiaram o uso de animais vivos, argumentando que o contato direto com material biológico proporciona uma experiência prática diferenciada e favorece a integração entre teoria, prática e pesquisa científica.

## Introduction

Biomedical research requires the use of live animals as natural or induced biomodels that replicate various diseases or pathological conditions, aiding in the study and understanding of pathogenesis, physiological mechanisms, and potential treatments (Luján et al., 2000). The use of experimental animals is arguably one of the foundational pillars in the advancement of health sciences and has enabled increasingly rapid progress in biological knowledge (Villamizar & Aquino, 2016). However, over time, concerns regarding animal welfare have grown, leading to the emergence of welfare-based movements and ethical frameworks, such as the 3Rs principle—Replacement, Reduction, and Refinement. This principle, introduced to students from the early stages of their undergraduate education, seeks to raise awareness and inform them about alternative methods to animal experimentation (Rawle, 2023).

The use of living animals in education has long been a subject of extensive debate. From the early days of in vivo experimentation and anatomical dissection aimed at understanding physiological systems, to the development of regulatory principles and the pursuit of alternatives recognizing animals as sentient beings with rights (Grimm et al., 2023), the conversation has evolved significantly. In the field of physiology, various teaching methodologies have been employed. For example, laboratory practices are considered by many authors to be ideal settings for learning about the physiology of organs and systems (Randall & Burkholder, 1990). Demonstrations using devices such as the physiograph, which measures physiological variables in live animals, offer “real material” and “real experience” that may enhance learning outcomes (Shore et al., 2013; Randall & Burkholder, 1990).

In contrast, non-invasive and animal-free methodologies are also available. These include computer-based laboratory simulations capable of mimicking organ system functions and physiological responses to stimuli (Richard et al., 1994); visual ethics laboratories focusing on the interaction between experimenter and subject (Senchina, 2011); and tools like LabTutor, a virtual laboratory offering structured modules that include background information, procedural guidance, real-time data recording, analysis, and report writing—most of which involve non-invasive procedures (Swift et al., 2016). These didactic tools support memorization of functional concepts, conceptual understanding, and experiential learning in physiology (Fernández & Madrid, 2015). Nevertheless, a central question arises: Is the use of living animals strictly necessary for learning?

Research into physiology education has grown in recent years, making it a fertile area for pedagogical exploration. Several studies have examined the implementation of innovative teaching strategies in both lectures and laboratory settings. One such strategy involves the use of didactic animal biomodels—constructed from inert materials—to simulate anatomical structures or physiological functions. These models, which have been employed since antiquity to understand physiological and pathological processes, serve as foundational tools for studies in human medicine (Gómez et al., 2015).

In Colombia, for instance, the use of biomodels and simulators in anatomy and physiology education has only recently gained traction (Lenis & Arango, 2011). Students are encouraged to apply their creativity, ingenuity, and research skills to design dynamic models that simulate specific physiological processes. This hands-on, step-by-step construction process enables students to build their own knowledge and explore biological functions across different species.

**Figure 1:**
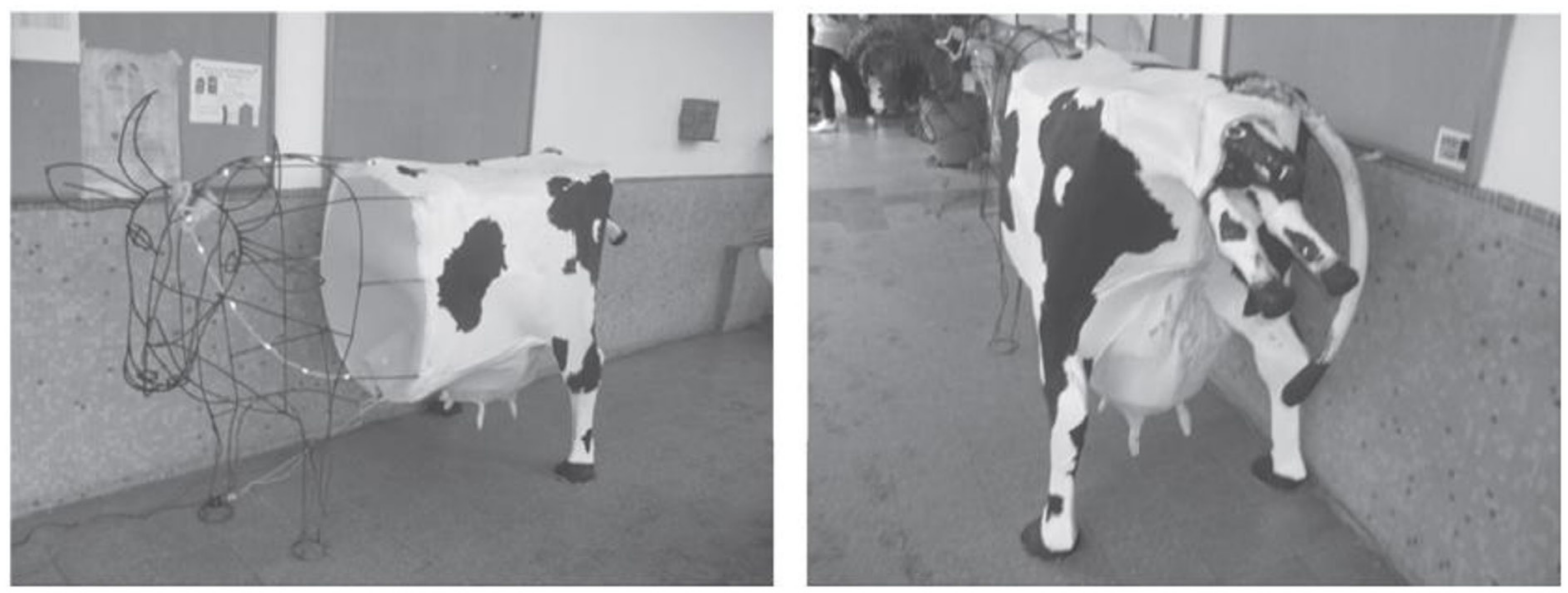
An example of a didactic biomodel built by veterinary medicine students at the University of Antioquia (Colombia) to exemplify bovine parturition. Taken from Lenis and Arango (2011).

Given this context, the key research question posed is: Could the implementation of didactic biomodels in an undergraduate veterinary physiology course stimulate student reflection on the necessity of using live animals in teaching and learning? To address this question, it is essential to explore students' experiences with biomodel construction and analyze their personal reflections on the pedagogical and ethical implications of using live animals in education (Figure 1).

## Materials and methods

The implementation of didactic biomodels and the exploration of the use of live animals in education were carried out with students in their second, third, and fourth semesters enrolled in the Physiology course during one academic year of the undergraduate program in Veterinary Medicine at the Agrarian Foundation University and the University of Applied and Environmental Sciences, both located in Bogotá, Colombia. In these courses, the final classroom project consisted of designing and constructing a didactic biomodel to represent a specific physiological process in either conventional or exotic species. To guide this process, an introductory session was conducted at the beginning of the semester to contextualize the development of the biomodels. Led by the course instructor, this session included a discussion on the historical background of biomodels, accompanied by visual materials such as photos and videos of previous student projects. A deadline was set for the end of the academic term. The completed biomodels were required to be both functional and pedagogical tools capable of clearly explaining a physiological topic to a general audience without relying on additional visual aids. As part of the learning experience, students were also shown uncensored audiovisual material depicting live animal experimentation. This content demonstrated how certain physiological discoveries, now widely accepted and featured in textbooks, were originally obtained. One example included the use of electromyography and the measurement of muscle potentials in frog legs, a practice that was common in physiology laboratories approximately 40 years ago. Once the biomodels were completed and presented, students were asked to provide experiential feedback and reflect on the use of live animals in the learning process. To this end, they were given an open-ended prompt: “After artificially recreating an invasive process that once involved live animals to gain physiological knowledge, would you consider the possibility of performing such experimentation on living animals during your education?” Students responded through a free-writing, open-expression format. For analysis, responses were first quantified to determine how many students agreed or disagreed with the use of live animals. Then, a qualitative analysis was performed to identify key themes across the narratives. This involved generating general thematic categories and grouping all responses—regardless of wording—that aligned with each category. For instance, if one student wrote “it would be very useful for learning,” and another wrote “it would be a tool for my education,” both responses were grouped under the category “learning.”

This analytical approach was informed by Laurence Bardin’s content analysis methodology (Bardin, 2011), a structured set of techniques for systematically interpreting verbal, written, or visual content to extract scientifically relevant insights. The analysis followed three key stages:

1. **Pre-analysis:** Organizing and preparing the content for examination, including a floating reading of the responses;
2. **Material exploration:** Coding the content and categorizing recording units to form thematic categories;
3. **Treatment of results, inference, and interpretation:** Relating the data to the theoretical framework and formulating conclusions—in this case, reflected in the categories presented in the following section.

## Results

**Figure 2:**
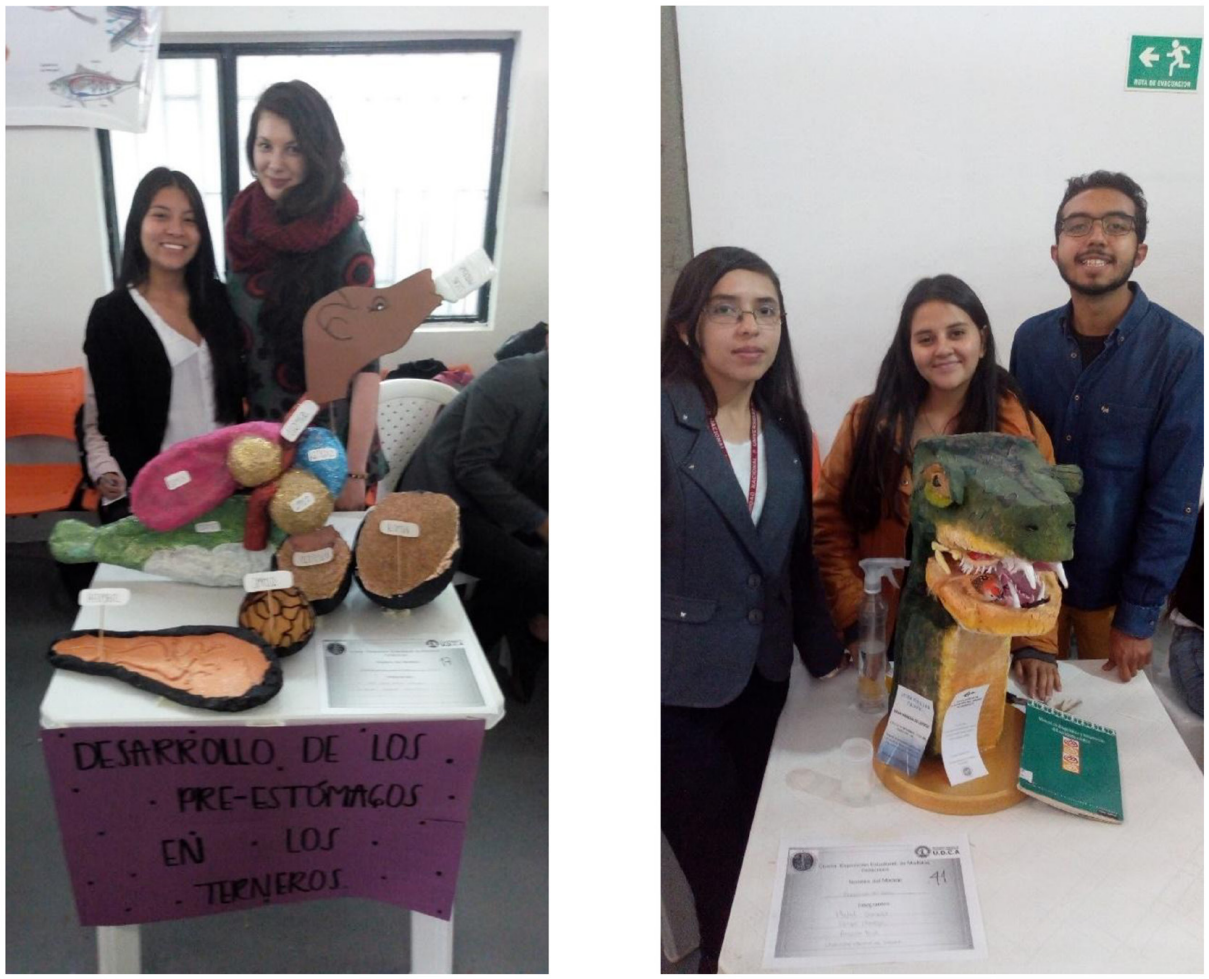
On the left image students represent the physiology of digestion in lactating ruminants in the digestive compartments with commonly used materials such as paper and probes. On the right image this biomodel explains the physiology of venom production in snakes by means of glands and sprays that simulate venom ejection.

**Figure 3:**
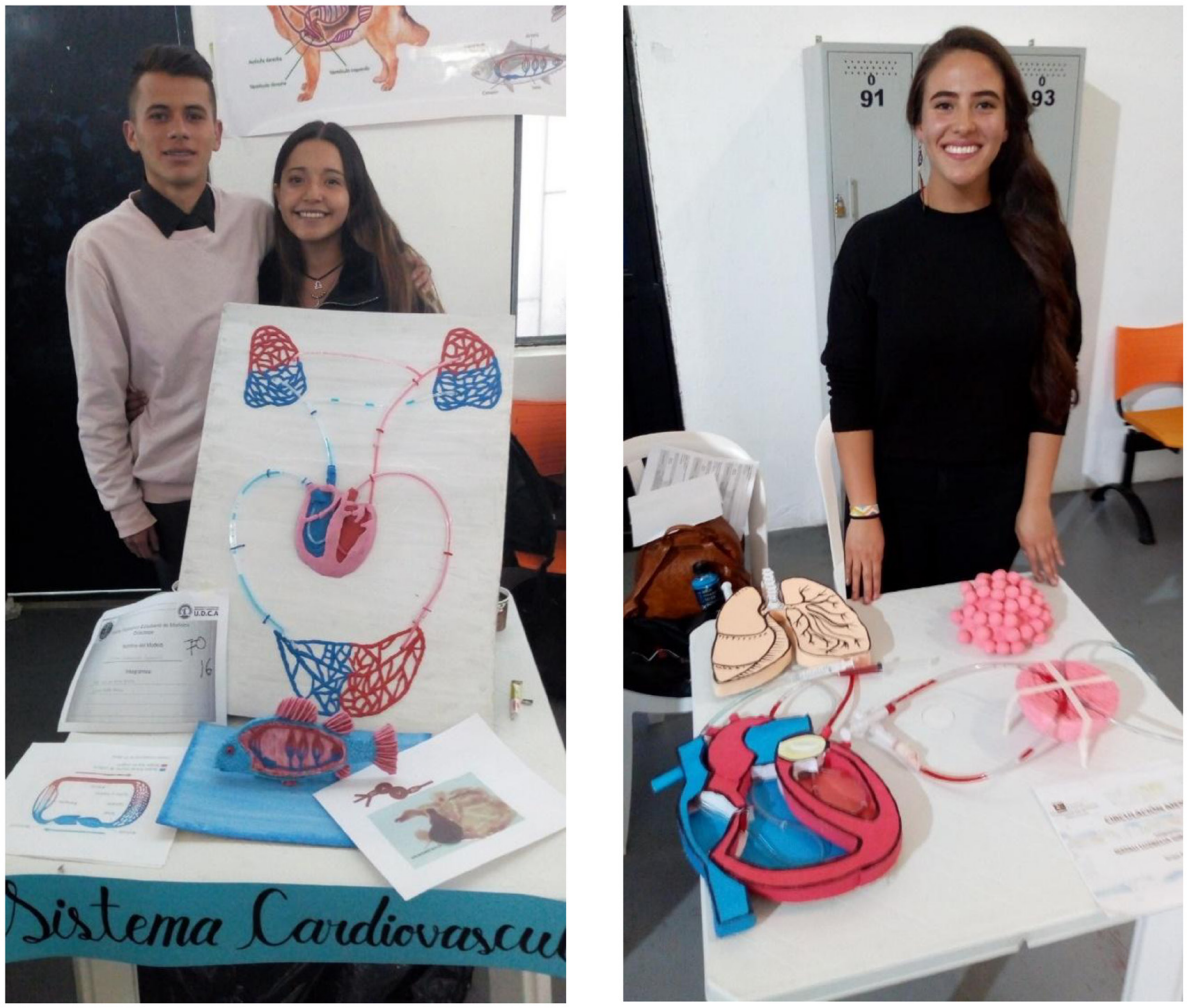
Students depicting cardio respiratory physiology along with alveolar and systemic gas exchange in fish and mammals. This image represents the exposure of primordial organs as lungs and heart to finally obtain the background of respiration.

**Figure 4:**
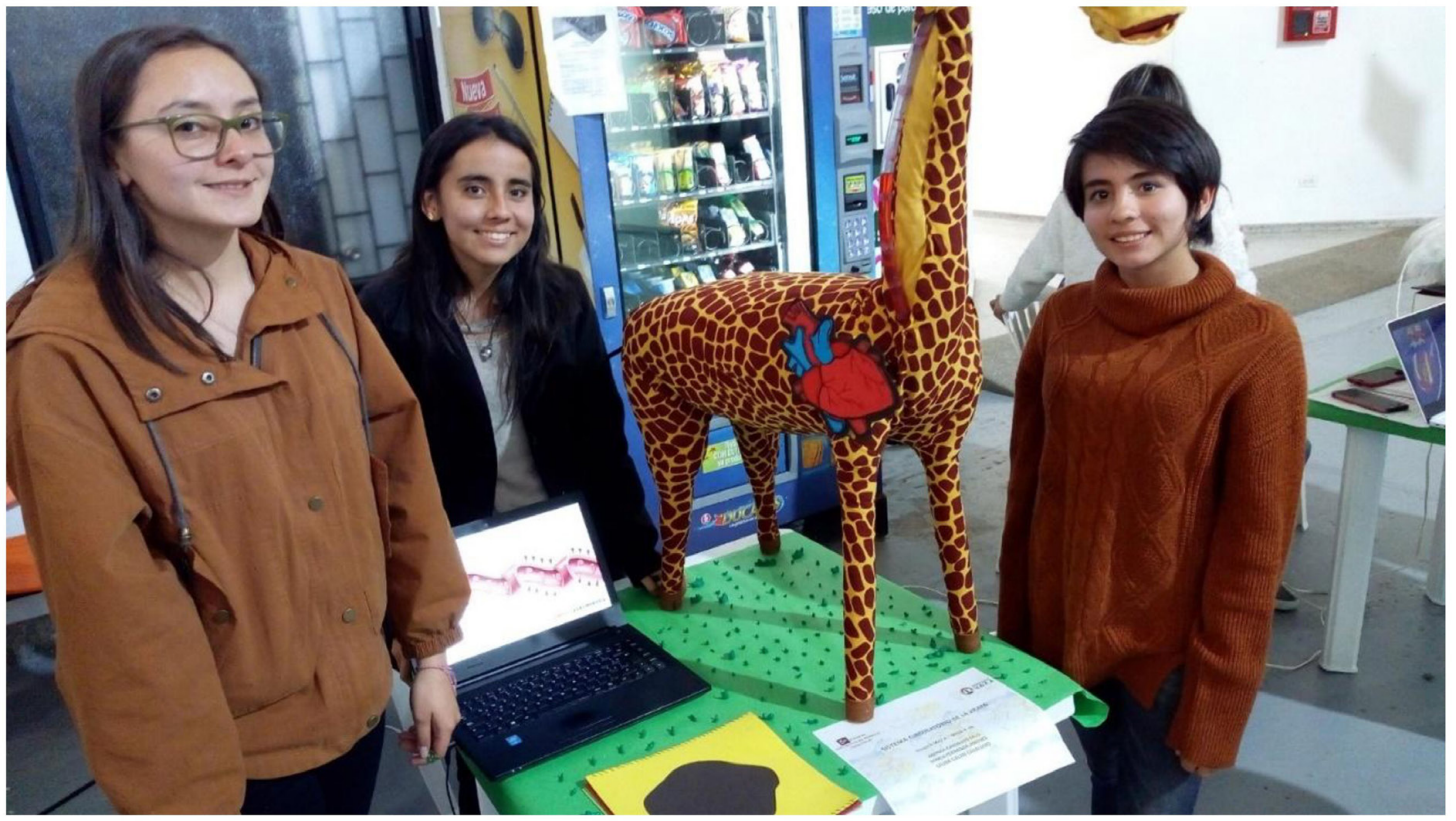
This is a biomodel on cardiac physiology in giraffes, the students made this model centered on the question: “*how is it possible for the giraffe to carry blood to the head on such a long neck*?”.

Photographic material of some physiology didactic biomodels by the students are presented (Figures 2, 3 and 4).

Following Bardin’s methodology, the analysis process was conducted in three structured steps. **In the first step (pre-analysis),** the students’ written reflections were organized to clearly distinguish the two positions—whether or not students would consider the use of live animals—as a basis for further analysis. **In the second step (exploration of the material)**, each text was summarized to extract the student’s final stance on the issue, identifying the rationale or justification behind their position. For example, one student wrote: “I do not agree with exploring the function of erythropoiesis at the cost of an animal’s life, since there are now alternatives such as simulators, molecular methods, and didactic materials that represent this already established knowledge.” This statement clearly reflects opposition to the use of live animals, grounded in the availability of alternative methods. **In the third step (treatment of the results: inference and interpretation)**, thematic categories were constructed based on the students’ viewpoints. These categories enabled the grouping of all narratives according to shared perspectives. The resulting categories included:

- **This practice provides experience**
- **Alternatives other than experimentation can be pursued**
- **It would not be done due to ethical principles and concern for animal welfare**
- **It serves as a learning tool**
- **It serves as an application of theoretical knowledge**
- **It is useful for research purposes**

In the example provided during the second step, the student’s response would be classified under the category “alternatives other than experimentation can be pursued.” This categorization process allowed for a structured, systematic interpretation of the data and served as the foundation for the thematic results presented in the following section.

The analysis experiential feedback data were grouped in Tables 1 and 2.

**Table 1:** Selection of participants regarding the use of live animals in physiology.

**POSSIBILITY TO USE LIVING ANIMALS AS BIOMODELS**	**NUMBER OF PARTICIPATING STUDENTS**	**PERCENT OF POPULATION**
**YES**	50	41.66%
**NO**	70	58.33%
**TOTAL**	120	100%

When quantifying “agreeing” or “disagreeing” the use of living animals it can be observed in that 58.33% of the participants do not accept the use of living animals while 41.66% accepted this this possibility.

**Table 2:** Reflection of participants regarding the use of live animals as biomodels to obtain knowledge in physiology.

**CREATED CATEGORY ACCORDING TO THE STORY**	**NUMBER OF PARTICIPATING STUDENTS**	**PERCENT OF POPULATION**
**EXPERTISE ADQUIRED**	8	6.66%
**ANOTHER ALTERNATIVES CAN BE USED**	37	30.83%
**NO FOR ETHICAL / ANIMAL WELFARE REASONS**	42	35%
**AS LEARNING METHOD**	26	21.66%
**APLICATION OF KNOWLEDGE**	3	2.5%
**AS A RESEARCH TOOL**	4	3.33%
**TOTAL**	120	100%

In the first place, the majority of students consider that there are alternatives other than experimentation with live animals. Secondly, a slightly lower percentage of students consider that this practice can serve as a way to obtain knowledge.

In the students’ arguments regarding the possibility of using invasive procedures on live animals for the creation of didactic biomodels in physiology, the majority rejected this option for ethical reasons, supported by concerns for animal welfare (35%). A significant portion of students believed that alternatives could be implemented to avoid the use of live animals (30.83%). Others accepted the possibility, stating that it provides a different classroom experience (6.66%) and enhances learning by offering access to “real material” (21.66%). A smaller group supported the use of live animals as a means of applying theoretical knowledge in practice (2.5%) or as a foundation for research (3.33%).

## Discussion

Historically, there has been a persistent reliance on animal experimentation to obtain scientific results—not only to uncover the mysteries of organismal biology but also for the sake of publication and professional recognition. As Molento and Calderón (2009) note, “*experimental results are often seen as more reliable than the obscure and contradictory statements of classical authors of antiquity*.” The publication of original findings has long been a source of pride among researchers—a tradition that continues to this day—and, in blunt terms, has cost the lives of thousands of animals in the pursuit of the physiological knowledge we now find in textbooks. When asked whether the sacrifice of so many living beings was necessary, the typical response might be: “*There was no other way to acquire this knowledge*.”

It is indisputable that many advances in physiological science have been achieved through animal experimentation, particularly in physiology, where understanding step-by-step biological processes often requires observation beyond what can be seen in dissected cadavers. Notable achievements include the work of Nobel laureates Santiago Ramón y Cajal (1852-1934) and Camillo Golgi (1843-1926), who were awarded the Nobel Prize in 1906 for their discoveries related to the structure and function of the nervous system; and Alexis Carrel (1873-1944), who in 1912 developed the isolated organ technique, keeping a chicken heart beating for months and pioneering vascular grafts and organ experiments (Molento & Calderón, 2009). This historical context raises a relevant question: *Is it still necessary to sacrifice animals in order to visualize phenomena that have already been described and documented in the scientific literature*? Even in the 18th century, James Ferguson (1760) proposed alternative methods to animal use, and Lazzaro Spallanzani (1729-1799) laid the foundation for in vitro experimentation (Molento & Calderón, 2009).

The use of animals in teaching has traditionally been valued for providing opportunities to observe living tissues and organ systems and to recognize physiological responses in real-time. In disciplines such as pharmacology, physiology, and anatomy, animal-based laboratory practices have long been considered essential. However, many of these educational activities suffer from poorly defined objectives, often justified by the need to teach practical skills such as handling, dissection, or surgery. This raises a critical ethical and pedagogical question: *If a student does not specialize in a particular field, and ultimately forgets the procedures involving live animals, was the sacrifice of those lives justified*? It is also commonly argued that such practices reinforce theoretical knowledge, teach data collection, and train students in analytical methods. But one must ask: Is the use of live animals truly the only way to achieve these educational goals? In many cases, equivalent outcomes may be reached using alternative, non-invasive methods. The European Convention for the Protection of Vertebrate Animals Used for Experimental and Other Scientific Purposes explicitly states that: “procedures on live animals shall be permitted only if the objectives cannot be achieved by audiovisual or other equally effective methods.” *Today, a wide array of simulators is available, ranging from cellular models to whole-system simulations”*.

In our study, students expressed a range of positions regarding the use of invasive procedures on live animals for biomodel construction in physiology. The majority rejected this possibility on ethical grounds and in support of animal welfare. It is plausible that, over time, social movements and educational exposure have influenced students’ perceptions, fostering the belief that animals are sentient beings with rights, thereby evoking empathy. Others believed that the growing availability of educational tools capable of simulating physiological processes—often with remarkable realism—renders the use of live animals unnecessary. These students may have independently concluded that it is illogical to sacrifice a living being to access information that can now be simulated repeatedly and non-invasively. Conversely, a minority of students supported the use of invasive procedures, largely based on the value of working with “real material.” It is unclear whether this preference stems from a personal conviction or is merely the reiteration of earlier pedagogical models that endorse animal sacrifice as essential for effective learning. Given that current generations have largely been educated in environments where live animal experimentation is minimal or absent, this view may be influenced by outdated paradigms or a perceived inadequacy of current alternatives to provide a “complete and authentic” experience. Nevertheless, this viewpoint remains valid and deserving of open and respectful discussion.

It is noteworthy that some students consider the use of animals to be justified for research purposes. This perspective may be influenced by the historical legacy of biomedical discoveries across medical disciplines. Many foundational developments in physiology, surgical techniques, pharmacodynamics, and related areas originated through animal experimentation—an origin story that is often highlighted by professors at the beginning of their courses. Another possible influence is the notion that results obtained through animal-based research can be extrapolated to human medicine for the benefit of patients.

A recurrent theme in classroom discussions is the perceived need for hands-on experience with animals in order to apply theoretical knowledge. This belief may stem from a desire to engage with real, tangible experiences that give purpose and context to the information acquired across different disciplines. Additionally, this view could be reinforced by the inherently practice-oriented nature of the veterinary medicine curriculum, which places a strong emphasis on academic activities that follow a sequence of theoretical instruction followed by practical application.

Within this pedagogical framework, constructivism plays a fundamental role in modern medical education. As a learning theory, it shifts the focus from passive reception of information (as in traditional teaching models) to active knowledge construction. In this model, the student is positioned at the center of the learning process, integrating new information with prior knowledge and experiences (Ortiz Cabrera, 2023). Furthermore, constructivism encourages critical skills such as analytical thinking, clinical reasoning, and decision-making—competencies that are essential in professional practice (Pinto et al., 2019). The educational exercise implemented in this study aimed to embody constructivist principles. By awakening students’ curiosity about the functioning of physiological systems in specific species, they were encouraged to research, retain, analyze, and evaluate relevant information. The culmination of this process was the design and construction of a didactic biomodel that was not only functional and pedagogical but also aesthetically effective. In doing so, the students actively participated in building their own knowledge, transitioning from theory to application through a creative, problem-solving process.

Didactic biomodels have long been used as educational tools. Their role in teaching—particularly within the natural, medical, and biological sciences—is crucial for facilitating meaningful, visual, and practical learning of abstract and complex concepts (Pesciallo et al., 2022). These models enable students to connect theoretical knowledge with tangible representations, thereby enhancing understanding in subjects such as anatomy, physiology, cell biology, and microbiology. According to Luján et al. (2000), biomodels offer an excellent opportunity for implementing activities based on research, collaboration, and problem-solving, all of which promote curiosity, objectivity, and the development of scientific reasoning. Lenis and Arango (2011) and Rodenbaugh et al. (2012) further argue that when students create biomodels themselves, the process fosters analytical, argumentative, and innovative skills. It also encourages creativity, imagination, and even entrepreneurship—qualities that can be especially beneficial in the teaching and learning of physiology. This pedagogical approach offers a potential shift away from the traditional one-way transmission of knowledge from teacher to student. Instead, it introduces an active, student-centered learning environment, which can also prompt deeper reflections—particularly regarding the ethical implications of performing invasive procedures on live animals. Such reflections are often shaped by the students’ personal experiences and the ideologies they choose to adopt or reject, often influenced by various societal or academic actors who either support or oppose these practices. In the present study, students’ thoughts and perspectives were explored without any form of coercion or influence. The creation of the biomodel served solely as a catalyst to elicit reflections that were likely already present in students' minds or that emerged organically during the process of model construction.

When comparing the findings of this study with those of other authors, several experiential similarities emerge regarding both the advantages of using didactic biomodels and students’ viewpoints on the use of animals in education. For example, Gookin et al. (2010), in their implementation of a didactic model to teach renal physiology, reported that most students found the model easy to understand, helpful in improving their knowledge, and effective in enhancing their appreciation for the filtration process. Notably, students expressed a preference for the model over traditional written materials and would recommend its use to peers. Similarly, Nayak and Soumya (2009), who developed a model to illustrate eye movements, found that all participating students considered the model highly beneficial for understanding the axes of movement. According to the researchers, the model was able to capture the students' attention within a minute. In another example, Anandit et al. (2018) created a muscle fiber model using simple materials such as ropes and balls to explain the physiology of muscle contraction. The three-dimensional nature of the model significantly aided students' understanding of skeletal muscle structure and function. Singh et al. (2018) implemented a model to illustrate the cardiac cycle. Students reported that it helped them remember key concepts, maintained their engagement, and made the session more interesting. Kuebler et al. (2007), using a model to teach respiratory mechanics, observed a 70% increase in student understanding compared to previous sessions taught solely through traditional lectures. Regarding the ethical debate on the use of live animals for educational purposes, similar findings were observed. Valverde et al. (2024) identified that students often adopt critical stances toward animal use in entertainment and education, rejecting such practices even when embedded in traditional or cultural contexts. Knight (2020) reports a growing preference among students for alternatives such as simulators, interactive videos, and digital models—preferences that have grown stronger as emerging technologies improve in quality and accessibility. Caballero García et al. (2018) also highlight positive ethical attitudes among students toward the non-use of animals, noting that increased educational exposure correlates with greater ethical awareness. Even within professional contexts, similar trends are evident. Reyes-Fernández et al. (2012) found that professionals acknowledge the importance of ethical regulations surrounding animal use. However, they also noted widespread normative ignorance, despite a consistent advocacy for animal welfare principles.

A central question shared by many educators is: How can we teach science in a meaningful and lasting way? We continuously strive to develop teaching methods that are comprehensive and capable of equipping learners with tools they can apply throughout their professional lives. While it is difficult to identify a single definitive educational method, one promising approach is active learning—a process that engages students in hands-on activities and encourages them to reflect on their ideas and how they apply them. Unlike traditional methods that emphasize the passive transmission of knowledge, active learning prioritizes student participation and fosters the development of critical skills such as scientific reasoning and complex thinking. This project was developed based on the idea of encouraging students to deviate from their traditional study routines by implementing an activity that emphasized individual responsibility in knowledge construction. The creation of biomodels served as a curricular complement within the physiology course, providing students with the opportunity to simulate physiological processes while engaging in discovery-based learning. These activities aimed to promote greater knowledge retention, deepen understanding, and cultivate a more positive attitude toward the subject matter. The theoretical foundation of this implementation was constructivism. Within this framework, the professor designs learning activities that enable the student to take control of their own learning process. Students are encouraged to build knowledge from multiple perspectives and at their own pace, integrating personal experience while simultaneously developing research and problem-solving skills. This approach also fosters a more focused and student-centered educational intervention, where learning becomes a dynamic process rooted in curiosity, creativity, and reflection.

## Conclusion

This type of educational exercise transforms the study of physiology into a space where theory, practice, and personal experience converge. Beyond the transmission of knowledge and the development of academic skills, it fosters the generation of new ideas, emotions, and ethical reflections. One such reflection—central to both this study and contemporary discourse in science education—is whether it is truly necessary to sacrifice animal lives in the name of learning. It is increasingly debatable whether students gain new insights through animal experimentation or whether they are simply repeating protocols whose outcomes are already well documented and unchanging. Moreover, the justification for using live animals may, in some cases, stem from traditional beliefs inherited from previous generations of educators, rather than from current pedagogical or scientific necessity. With the advancement of technology and educational resources, it is now possible to create reproducible, scalable models that can effectively replace the use of animals in undergraduate veterinary education. These models not only provide students with practical and theoretical understanding but also support long-term memory retention and professional development. In this project, we were able to explore students’ perspectives on the use of living animals in the acquisition of physiological knowledge. As educators, we witnessed their enthusiasm, curiosity, and deep engagement in the creation of didactic biomodels. Most significantly, we observed a shift in learning dynamics, as students took responsibility for their own education and demonstrated a growing sense of ownership and empowerment over their knowledge.
